# Long-Range Effects of Wing Physical Damage and Distortion on Eyespot Color Patterns in the Hindwing of the Blue Pansy Butterfly *Junonia orithya*

**DOI:** 10.3390/insects9040195

**Published:** 2018-12-19

**Authors:** Joji M. Otaki

**Affiliations:** The BCPH Unit of Molecular Physiology, Department of Chemistry, Biology and Marine Science, Faculty of Science, University of the Ryukyus, Nishihara, Okinawa 903-0213, Japan; otaki@sci.u-ryukyu.ac.jp; Tel.: +81-98-895-8557

**Keywords:** butterfly wing, color pattern, damage, distortion, eyespot, hindwing, long-range signal, morphogenic signal

## Abstract

Butterfly eyespot color patterns have been studied using several different approaches, including applications of physical damage to the forewing. Here, damage and distortion experiments were performed, focusing on the hindwing eyespots of the blue pansy butterfly *Junonia orithya*. Physical puncture damage with a needle at the center of the eyespot reduced the eyespot size. Damage at the eyespot outer rings not only deformed the entire eyespot, but also diminished the eyespot core disk size, despite the distance from the damage site to the core disk. When damage was inflicted near the eyespot, the eyespot was drawn toward the damage site. The induction of an ectopic eyespot-like structure and its fusion with the innate eyespots were observed when damage was inflicted in the background area. When a small stainless ball was placed in close proximity to the eyespot using the forewing-lift method, the eyespot deformed toward the ball. Taken together, physical damage and distortion elicited long-range inhibitory, drawing (attracting), and inducing effects, suggesting that the innate and induced morphogenic signals travel long distances and interact with each other. These results are consistent with the distortion hypothesis, positing that physical distortions of wing tissue contribute to color pattern determination in butterfly wings.

## 1. Introduction

An important research topic in developmental biology is the characterization of physiological functions and mechanisms of organizers for developmental fate determination. An organizer, discovered first in amphibian embryos using surgical manipulations, releases a signal called a morphogen to determine the developmental fate of immature cells [1,2,3,4]. Since the original surgical study of Spemann and Mangold (1924) [1], at least four regions of embryos have been identified as organizers in vertebrates [4]. 

Surgical manipulations have played an important role in butterfly biology. Cautery or puncture damage studies and surgical transplantation studies on nymphalid butterfly wings have demonstrated that the center of the prospective eyespot in a pupal forewing tissue functions as an organizer; an application of cautery or simple physical puncture damage to the center of the prospective eyespot reduced the eyespot size in adult wings [5,6,7,8,9,10,11], and transplantations of the center of the prospective eyespot induced an ectopic eyespot around the transplant [5,6,12]. These two lines of evidence established that the center of an eyespot functions as an organizer and a source of morphogen for color pattern determination. Additionally, damage responses in the background area (where no color pattern element is located) [6,10,11,13,14] and physiologically induced color pattern changes [6,15,16,17,18,19,20] have been investigated to understand the nature of the putative morphogenic signals and the immature scale cells that receive and interpret the signals.

As an experimental system, the butterfly wing system has several technical advantages. Butterfly wings are mostly two-dimensional, although they are three-dimensional in a rigorous sense [21], and thus, suitable for identifying normal and induced fates. Eyespot organizers are easily identifiable at the pupal stage, because they are located immediately beneath the pupal cuticle spots [9,21]. Live organizing cells have been observed in vivo as nadir cells at the bottom of the focal indentation in the ventral forewing [22]. Butterflies have right and left wings, and their color patterns are almost always identical, which means that the internal control is always available when only the right or left wing is subjected to experimental manipulation [23]. The results are visually compelling, as the color pattern changes, and thus, easy to interpret after experimental manipulation. Color fates are limited in a small number of colors, and there is the one-cell one-scale rule, meaning that a given scale cell produces just a single scale of a discrete color [6,24], which makes the interpretation at the cellular level simple.

However, the mechanism for color pattern determination has been enigmatic. Classically, the putative morphogen for eyespot color patterns was thought to establish a static concentration gradient of the morphogen, and based on the local concentration, each immature cell determines the cell’s fate [6,7,25,26,27] according to the concentration gradient model for positional information [28,29,30]. However, it has been noted that the classical gradient model cannot explain a variety of real nymphalid eyespots as opposed to the ideal concentric eyespot [31]. Importantly, parafocal elements (PFEs) have been shown to be remote, outermost eyespot rings [31,32,33]; eyespots and PFEs are collectively called the border symmetry system in the nymphalid ground plan [6,24,34,35]. Accordingly, a model that explains both eyespots and PFEs has been proposed [24,36,37,38]. Experimentally, physical damage to the forewings of the peacock pansy butterfly *Junonia almana* revealed that two adjacent eyespots interact with each other in an inhibitory or activating manner and that damage to the background area induces an ectopic organizer for eyespot-like structures [10,11]. These experimental results have been incorporated into the induction model, which proposes dynamic morphogenic signal interactions for color pattern determination [24,31,37].

However, information on the damage response has been accumulated mostly from forewings, and information from hindwings is limited. There is a possibility that hindwing responses to physical damage are different from those of the forewing in *Junonia coenia* [6], although this was not the case with *J. almana* [11]. In addition, the previous damage experiment was performed mostly to test the function of the central cells at the prospective eyespot as an organizer but not to examine the possible long-range effects between the damage-induced signals and the innate eyespot signals. Because physical distortions may play an important role in color pattern formation in butterfly wings, according to the distortion hypothesis for morphogenic signals [24,39], hindwing responses are expected not only from simple physical damage but also from physical distortions. 

**Figure 1:**
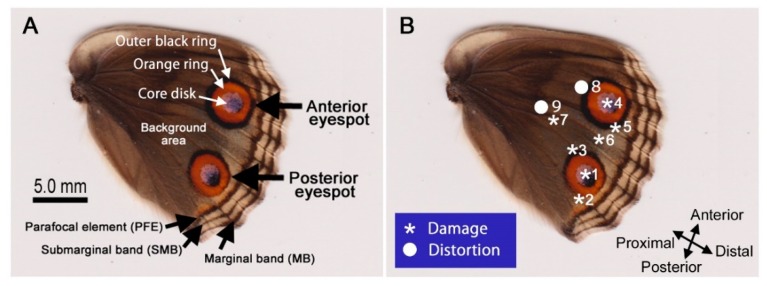
The hindwing of *Junonia orithya*. (**A**) Nomenclature of the hindwing elements and sub-elements. (**B**) Treatment modes. The physical puncture damage sites (Modes 1–7 in asterisks) and ball placement sites (Modes 8 and 9 in white circles) are indicated by numbers of treatment modes. Modes 7 and 9 include the broader background area.

Here, I performed damage and distortion experiments, focusing on the hindwing of the blue pansy butterfly *Junonia orithya*. The hindwing of this species has two large eyespots against the background area without any other complicated patterns except the peripheral bands (i.e., PFEs, submarginal bands, and marginal bands) (Figure 1A). Thus, this system is ideal for observing interactions between experimental stimuli and innate eyespot signals. 

## 2. Materials and Methods 

### 2.1. Butterflies

In this study, *J. orithya* (Linnaeus, 1758) was used as an experimental model. Only female individuals were examined after treatment because of sexual dimorphism in this species; females have large anterior and posterior eyespots on their hindwings with a brown or blue background area (Figure 1A), whereas males have a compromised anterior eyespot (or black spot) and a relatively small posterior eyespot on their hindwings with a metallic blue background area.

Females of this species were field-collected from the Nishihara Campus of University of the Ryukyus, Okinawa, Japan in 2015–2018. Eggs were harvested from these females on the surface of host plant leaves in a 300 mm × 300 mm × 300 mm frameless glass tank called a Crystal Cube 300 (Kotobuki Kogei, Tenri, Nara, Japan) at ambient temperatures of approximately 27 °C. They were fed ad libitum with a commercially available “ion supply drink”, POCARI SWEAT (Otsuka Pharmaceutical, Tokyo, Japan).

The larvae were reared with natural host plant leaves native to Okinawa-jima Island, *Phyla nodiflora* or *Plantago asiatica*. In addition, *Hemigraphis alternata* was occasionally given when the former two plants were not available, because the blue pansy butterfly on Okinawa-jima Island appeared to use this plant in the wild in my observation. These host plant leaves were wild harvested mainly from the Nishihara Campus of the University.

### 2.2. Experimental Operations

Two treatment modalities were performed: physical puncture damage with a needle (Modes 1–7) and ball placement on the hindwings after the forewing-lift operation (Modes 8 and 9). The effect of the transient forewing-lift procedure (no ball placement between the wings) was also tested (Mode 10). The treatment was performed only on the right wing. The left wing was left intact for comparison. Each individual was treated by a single operation throughout this study.

For the physical puncture damage experiments (Modes 1–7), a stainless needle of 0.50 mm in diameter (Shiga Konchu, Tokyo, Japan) was used to damage the wing tissue within 6 hours after pupation. The site to be damaged was visually navigated and identified based on the surface patterns of the pupal wing cuticle (i.e., pupal cuticle spots). The needle was pushed down approximately 3 mm in depth and moved up and down slowly 10 times.

The treatment modes that were performed are summarized as follows (Figure 1B): physical damage at the center of the posterior eyespot (Mode 1), physical damage at the posterior side of the posterior eyespot (Mode 2), physical damage at the proximal side of the posterior eyespot (Mode 3), physical damage at the center of the anterior eyespot (Mode 4), physical damage at the posterior side of the anterior eyespot (Mode 5), physical damage at the background area between the anterior and posterior eyespots (Mode 6), and physical damage at the proximal background area (Mode 7). 

For the ball placement (distortion) experiments (Modes 8 and 9), a stainless ball (Tsubaki Precision Balls) of 0.50 mm in diameter (Tsubaki Nakashima, Katsuragi, Nara, Japan) was used to produce wing tissue distortion within 30 min postpupation. A ball was placed at the anterior side (Mode 8) of the right hindwing after the forewing-lift procedure, and the lifted forewing was placed back into the original position. The ball was thus sandwiched between the forewing and hindwing. A ball was also similarly placed at the proximal side of the hindwing where no color pattern element was located (Mode 9). For comparison, the forewing was lifted transiently, and within 1 min, the lifted forewing was placed back into the original position (Mode 10).

These treatment modes are summarized as follows (Figure 1B): ball placement at the proximal side of the anterior eyespot (Mode 8), ball placement at the proximal background area (Mode 9), forewing-lift control (no ball placement) (Mode 10), and no treatment (Mode 11).

### 2.3. Data Analysis

Because the color patterns of this species are highly variable among individuals, even in females, the color patterns of the treated right hindwing were always compared with those of the non-operated left hindwing of a given individual. The eyespot size change was visually evaluated based on the core disk size of the treated right hindwing in comparison with that of the left hindwing without treatment. The outer black ring and orange ring were not considered for eyespot size evaluation, because they were easily drawn to neighboring damage or distortion, whereas the black core disk appeared to be less sensitive. Similarly, minor deformation that did not contribute to the size change was not considered to be size modification. However, phenotypic traits that were different from the left hindwing in an individual were descriptively recorded. Statistical analysis (*χ*^2^ test or Fisher’s exact test) was performed using JSTAT (Yokohama, Japan) when necessary. For *χ*^2^ tests, statistical values after Yates correction were reported.

## 3. Results

### 3.1. Physical Puncture Damage at the Posterior Eyespot

**Figure 2:**
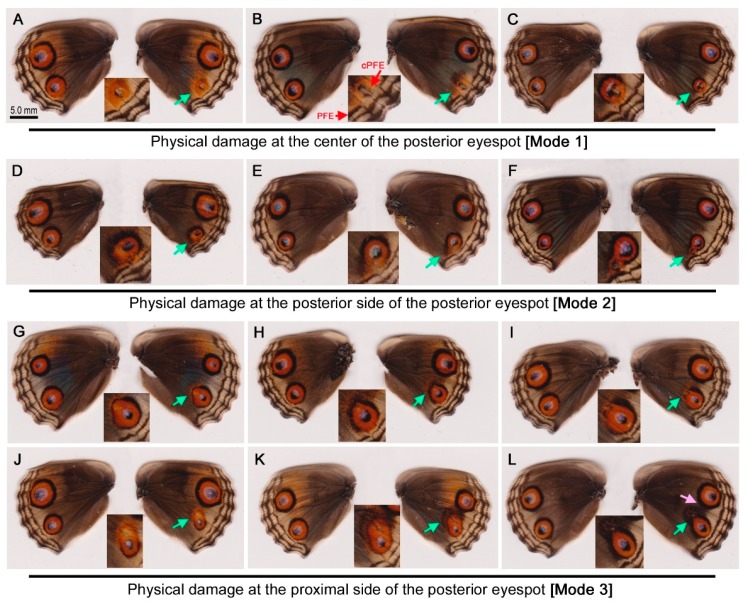
Color pattern modifications induced by physical damage at the posterior eyespot in *J. orithya*. In each individual, the right wing was operated on, and the left wing was left intact for control purposes. The green arrows indicate the sites of the puncture damage and the induced patterns around those sites. The insets are enlargements of the modified eyespots. (**A**–**C**) Damage at the center (Mode 1). The eyespot was reduced in size. In B, the parafocal element (PFE) in the operated compartment was also reduced in size and dislocated toward the center of the eyespot, indicated as compromised PFE (cPFE). (**D**–**F**) Damage at the posterior side (Mode 2). In (**D**,**E**), the posterior eyespot became smaller. In (**E**), the outer black ring was open. In (**F**), an ectopic eyespot was produced, and the innate eyespot was drawn to and fused with the ectopic eyespot. (**G**–**L**) Damage at the proximal side (Mode 3). In most individuals, the eyespot was deformed and reduced in size. The entire eyespot was drawn to the damage site despite the size reduction. In contrast, in (**L**), the entire posterior eyespot was elongated toward the damage site, resulting in a slight increase in the eyespot size. The pink arrow indicates an additional modified site of the innate eyespots (the fusion of the outer black ring of the anterior eyespot with the induced black area).

In response to physical damage at the center of the posterior eyespot (Mode 1), the treated individuals showed a reduction in the size of the treated eyespot without exception (*n* = 4) (Figure 2A–C). The anterior eyespots showed no change. Interestingly, in a treated individual, the PFE in the proximity of the treated eyespot moved toward the center of the reduced eyespot (Figure 2B), although this compromised PFE (cPFE) was not observed in other individuals (Figure 2A and C).

In response to physical damage at the posterior side of the posterior eyespot (Mode 2), the eyespot slightly reduced (*n* = 7) or slightly enlarged (*n* = 3) (Figure 2D–F). The reduction (Figure 2D, E) may have been because the damage operation wrongly damaged the organizing cells at the center of the prospective eyespot. However, the noncentral damage might have influenced the eyespot size without physically damaging the organizing cells. The slight enlargement (Figure 2F) may have been because of fusion with the ectopic eyespot induced by the treatment. 

In response to physical damage at the proximal portion of the posterior eyespot (Mode 3), the majority of the treated individuals showed a reduction of the posterior eyespot core disk (*n* = 23) (Figure 2G–K) despite a notable distance from the damage site to the core disk. In some of these individuals, the longitudinal direction of the eyespot changed toward the damaged site. Some individuals showed eyespot enlargement (*n* = 3) (Figure 2L) or no change (*n* = 9). In 3 cases, the black area induced by the treatment fused with the outer black ring of the anterior eyespot (Figure 2L), in which case the outer black ring of the anterior eyespot was thickened. However, in the other 20 cases, the anterior eyespot showed no change.

### 3.2. Physical Puncture Damage at the Anterior Eyespot

**Figure 3:**
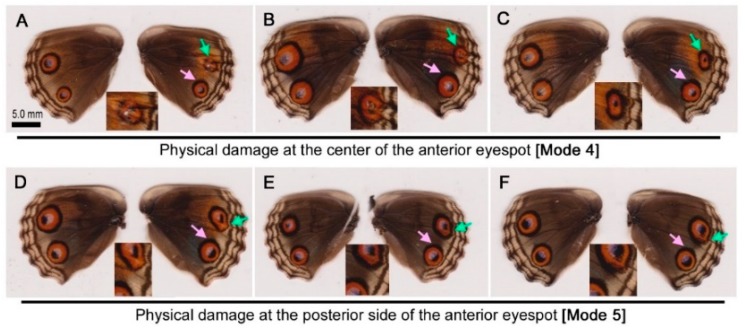
Color pattern modifications induced by physical damage of the anterior eyespot in *J. orithya*. In each individual, the right wing was operated on, and the left wing was left intact for control purposes. The green arrows indicate sites for puncture damage and the induced patterns around those sites. The pink arrows indicate additional modified sites of the innate eyespots. The insets are enlargements of the modified eyespots. (**A**–**C**) Damage at the center (Mode 4). The eyespots were reduced in size. In response to this treatment, the posterior eyespot core disk was enlarged (**A**) or slightly reduced (**B**,**C**). However, in all the cases shown, the outer black ring of the posterior eyespot was thickened at the side facing the anterior eyespot. (**D**–**F**) Damage at the posterior side (Mode 5). In all the cases shown, the anterior eyespot was deformed, and the outer black ring of the posterior eyespot facing the anterior eyespot was thickened (pink arrows).

In response to physical damage at the center of the anterior eyespot (Mode 4), the treated individuals showed eyespot reduction in size without exception (*n* = 7) (Figure 3A–C). Interestingly, the posterior eyespot responded by thickening the outer black ring in 4 individuals with treated wings (Figure 3A–C). Among these, posterior eyespot enlargement together with the outer black ring was observed (*n* = 1) (Figure 3A). However, in 3 individuals, the posterior eyespot core disk slightly reduced in size despite the expansion of the outer black ring (Figure 3B, C).

In response to physical damage at the posterior side of the anterior eyespot (Mode 5), the majority of the treated individuals showed eyespot reduction (*n* = 9) (Figure 3D). However, enlargement (*n* = 1) (Figure 3E) and no size change but with deformation (*n* = 2) were observed (Figure 3F). Among these 12 individuals, the posterior eyespot responded by thickening the outer black ring in 8 individuals despite a notable distance from the center of the eyespot (Figure 3D–F). 

To confirm that this asymmetric thickening (i.e., drawing or attracting) effect did not originate from natural asymmetric variation, hindwings of no-treatment individuals (Mode 11; see below) were examined (*n* = 51). Only one individual showed an asymmetric enlargement of the outer black ring of the posterior eyespot that was somewhat similar to the experimental results. The experimental and no-treatment frequencies of such modifications were significantly different (Fisher’s exact test, *p* < 0.0001). This means that the drawing effects were likely caused by the physical puncture damage at the posterior side of the anterior eyespot.

### 3.3. Physical Puncture Damage at the Background Area

**Figure 4:**
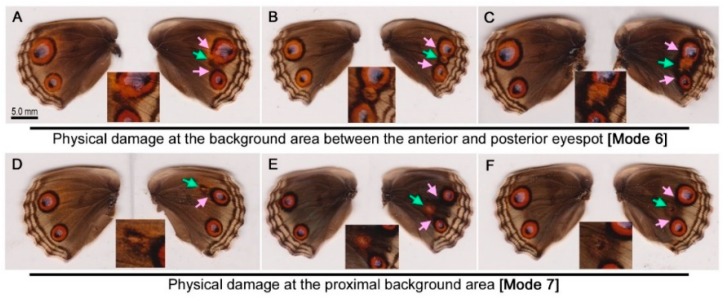
Color pattern modifications induced by physical damage to the background area in *J. orithya*. In each individual, the right wing was operated on, and the left wing was left intact for control. The green arrows indicate sites of puncture damage and the induced patterns around those sites. The pink arrows indicate additional modified sites of the innate eyespots. The insets are enlargements of the modified sites. (**A**–**C**) Damage between the two eyespots (Mode 6). In (**A**), the outer black ring was broken, and the entire anterior eyespot was deformed. In (**B**,**C**), ectopic eyespot-like structures were produced and fused with the anterior and posterior eyespots. (**D**–**F**) Damage at the proximal background area (Mode 7). In addition to the induced elements, the outer black ring of the innate eyespots is drawn toward the damage site.

Physical damage of the background area between the anterior and posterior eyespot (Mode 6) induced a ring break (*n* = 4) (Figure 4A), an ectopic eyespot-like structure fused with two innate eyespots (*n* = 4) (Figure 4B, C) or a black area (*n* = 2). 

Physical damage to the proximal background area (Mode 7) induced a small orange area enclosed by a black area (*n* = 5) (Figure 4D,E) or a relatively large black area with or without an orange area (*n* = 3) (Figure 4F). Among these, fusion or a drawing effect of the outer black ring of either anterior or posterior eyespot was observed in 6 cases despite a notable distance between the damage site and the eyespot (Figure 4D–F). 

### 3.4. Ball Placement

**Figure 5:**
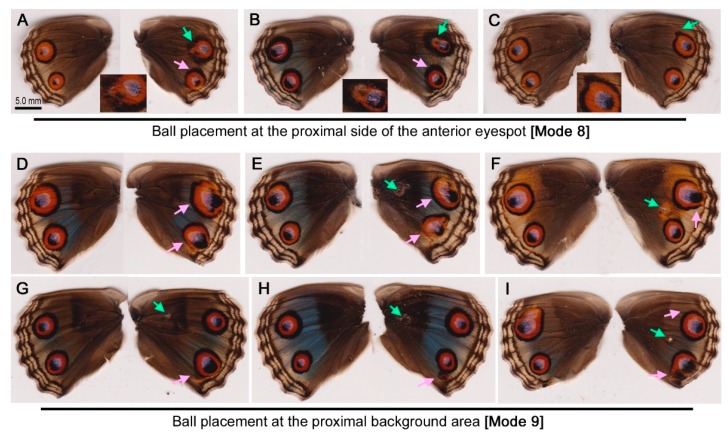
Color pattern modifications induced by ball placement and a transient forewing-lift operation in *J. orithya*. In each individual, the right wing was operated on, and the left wing was left intact for control purposes. The green arrows indicate sites for puncture damage or ball placement. The pink arrows indicate modified sites. (**A**–**C**) Ball placement at the proximal side of the anterior eyespot (Mode 8). The insets are enlargements of the modified eyespots. (**D**–**I**) Ball placement at the proximal background area (Mode 9).

Ball placement at the proximal side of the anterior eyespot (Mode 8) was performed. In this treatment (Mode 8), the ball placement site was close to or inside the anterior eyespot. Among the 10 individuals tested, no change was observed in 5 individuals. The other 5 individuals had some changes in either or both eyespots (Figure 5A–C) including anterior eyespot enlargement and deformation (*n* = 1) (Figure 5A), reduction and deformation (*n* = 1) (Figure 5B), and deformation without a size change (*n* = 3) (Figure 5C). In these cases, the anterior eyespot deformation toward the ball was characteristic pf this treatment. Among these, one individual showed both anterior and posterior eyespot enlargement (Figure 5A) and another individual showed a reduced and deformed anterior eyespot and an enlarged outer black ring of the posterior eyespot (Figure 5B). In these 2 individuals, the outermost black ring was thickened, possibly toward the ball insertion point. The posterior eyespots showed no change in 3 cases that had minor changes in the anterior eyespot (Figure 5C).

To confirm that these asymmetric modifications induced by the ball placement above did not originate from natural asymmetric variation, the hindwings of the forewing-lift individuals (Mode 10; see below) were examined (*n* = 30). No individuals showed a similar asymmetric modification, and the results of these two modes were significantly different (Fisher’s exact test; *p* = 0.0004). This means that the ball placement likely caused the color pattern changes.

Ball placement on the background area (the proximal portion of the hindwing) (Mode 9) was then performed. In this treatment (Mode 9), the ball placement site was relatively far from the eyespots, being different from the treatment above (Mode 8). No change was observed throughout the wings of 27 individuals, but some were modified (*n* = 22) (Figure 5D-I), resulting in a modification rate of 44.9%. However, the modification profiles were as follows: enlargement (*n* = 6), reduction (*n* = 2), and no change (*n* = 14) in the anterior eyespot; and enlargement (*n* = 17), reduction (*n* = 1), and no change (*n* = 4) in the posterior eyespot. Five individuals showed some changes in both anterior and posterior eyespots (Figure 5D, E, I). Among them, both eyespots were enlarged (Figure 5D,E), except in one individual (Figure 5I), where the large innate fusion eyespot was reduced, probably because the most anterior eyespot organizer was inhibited due to the operation.

### 3.5. Forewing-Lift Control

**Figure 6:**
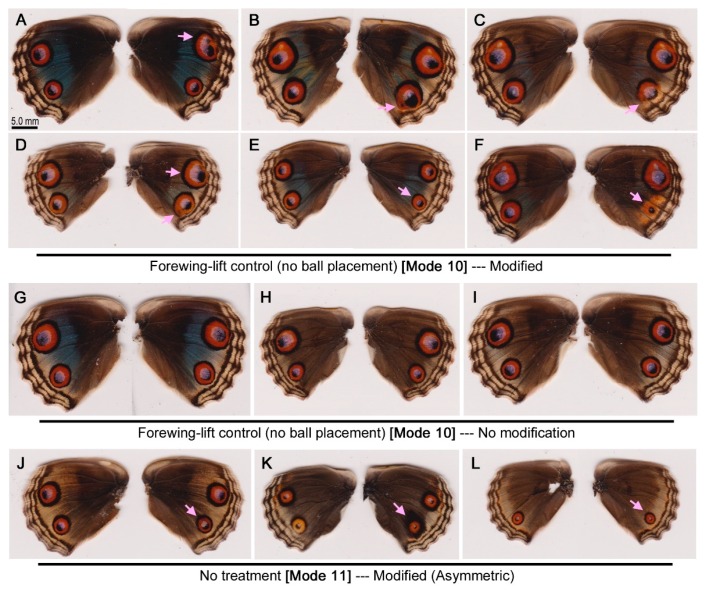
Color pattern modifications induced by the forewing lift and no treatment controls in *J. orithya*. In each individual, the right wing was operated on, and the left wing was left intact for control purposes. The pink arrows indicate modified sites. (**A**–**F**) Forewing-lift operation (Mode 10). Only modified individuals are shown here. (**G**–**I**) Forewing-lift operation (Mode 10). Only nonmodified individuals are shown here. (**J**–**L**) No treatment (Mode 11). Rare individuals with asymmetric color patterns are shown here.

The wing-wide changes that were observed in the ball placement experiments above were tested to determine if they were induced by the transient forewing-lift procedure without ball placement (Mode 10). In total, 30 individuals were examined. Surprisingly, 12 individuals showed some modifications (Figure 6A–F), and no modifications were observed in either the anterior or posterior eyespots in 18 individuals (Figure 6G–I), resulting in a modification rate of 40.0%. No individuals had reduced anterior eyespots, but anterior eyespots were enlarged in 2 individuals (Figure 6A,D). On the other hand, 6 individuals showed enlarged posterior eyespots (Figure 6B–D), and 2 showed reduced posterior eyespots (Figure 6E,F).

The hindwings of individuals with no treatment were also examined for comparison (Mode 11). Among 51 individuals, only 3 individuals had color pattern differences between the right hindwing and the left hindwing (Figure 6J–L), resulting in a modification rate of 5.6%.

Each modification should be evaluated in comparison to the contralateral, nontreated left wing due to individual variation, but frequencies of modifications can be tested statistically to evaluate different modes of operation. The possible difference between the modified and nonmodified individuals (between Modes 9 and 10) was compared using the *χ*^2^ test, resulting in no significance (*df* = 1, *t* = 0.037, *p* = 0.32). This means that the ball placement at the proximal background area did not induce any change. When Modes 10 and 11 were compared, the difference was significant (*df* = 1, *t* = 13.3, *p* = 0.0003). This means that the forewing-lift procedure itself induced changes. When Modes 9 and 11 were compared, the difference was also significant (*df* = 1, *t* = 19.5, *p* <0.0001). Together with the previous results, this also means that the forewing-lift procedure itself caused the color pattern changes.

## 4. Discussion

### 4.1. Hindwing Damage Experiments

This study focused on the hindwing eyespots of the blue pansy butterfly, *J. orithya*. Previous damage studies have focused on the forewing [5,6,7,8,9,10,13,14], except a brief description of *J. coenia* [6] and a recent study of *J. almana* [11], probably because the forewing is located on the surface of a pupa and, thus, easy to manipulate. However, at least in *J. orithya*, the hindwing color pattern is simpler than that of the forewing, and therefore, the hindwing deserves the attention of damage studies. 

Previously, Nijhout (1991) [6] briefly described a hindwing damage experiment using *J. coenia* without a rigorous method and data presentation, mentioning that the eyespot damage did not elicit any changes but the background damage induced an ectopic eyespot. It was not until Iwasaki and Otaki (2017) [11] that the hindwing damage response was systematically studied using *J. almana*, demonstrating that the hindwing eyespot responded to damage similarly to the forewing eyespot. However, the major hindwing eyespot of *J. almana* is a large fusion of two eyespots, which makes it unsuitable for studying damage response profiles of simple eyespots. In this sense, the present study is the first damage study to focus on simple hindwing eyespots in butterflies.

The central damage to the eyespot reduced the eyespot size in both anterior and posterior eyespots in the hindwing of *J. orithya* (Figure 2A–C and Figure 3A–C), suggesting that organizing cells are located at the center of the hindwing eyespot, which functions as a morphogen source, consistent with Iwasaki and Otaki (2017) [11]. The present results are in contrast to Nijhout (1991) [6], who suggested a sink (instead of source) of morphogen at the center of an eyespot using *J. coenia*. If so, this discrepancy may originate from fundamental mechanistic differences in having a morphogen source or sink between the two species.

An alternative explanation is that the discrepancy stemmed from developmental heterochrony. In *J. almana*, late damage at the center of a forewing or hindwing eyespot enlarged the eyespot [10,11] as if the center of the prospective eyespot functioned as a morphogen sink. The late damage response in *J. almana* can be explained as a summation of the innate and experimentally induced signals, because the organizer is no longer active at the time of damage [10,11]. The *J. coenia* hindwing may have a developmental time course different from that of the *J. almana* or *J. orithya* hindwing. If so, any damage after pupation may be too late to induce any changes in eyespots.

However, another explanation is that the cautery treatment in Nijhout (1991) [6] was simply misplaced, for inflicting precise damage at the organizer is difficult in the hindwing. In the case of the *J. almana* damage experiment, some individuals who were subjected to central eyespot damage did not show any changes, probably because of a misplacement of damage [11].

### 4.2. Long-Range Effects

Damage at the eyespot outer black ring or its proximity not only deformed the entire eyespot toward the damage site, but also diminished the core disk despite a notable distance to the central area; the eyespot signal was drawn toward the damage site, and simultaneously, the core disk signal was inhibited (Figure 2D,E,G–L and Figure 3D–F). Thus, noncentral damage to an eyespot may be able to cause dysfunction of the existing organizing cells in *J. orithya*. The damage-induced inhibitory signals that traveled long distances likely repressed the function of the organizing cells. However, because the damage site was relatively close to the central area, the possibility that the core disk area was directly damaged in error during the damage operation for the outer rings cannot be excluded completely.

Interestingly, when damage was inflicted to the posterior side of the anterior eyespot, the outer black ring of the posterior eyespot was thickened and drawn toward the damage site (Figure 3D–F). In this case, the damage site was far apart from the posterior eyespot, excluding the possibility that the posterior eyespots were directly damaged in error. Indeed, the posterior eyespot core disk size did not change. Similarly, damage at the middle of the two eyespots induced fusion of the two innate and one induced eyespots (Figure 4A–C), and the damage to the proximal background area drew the anterior and posterior eyespots toward the damage site (Figure 4D–F). These results showed that not only the induced signals, but also the innate signals are long ranging. The different behavior of the outer parts (the outer black and orange rings) and the core parts (the core disk) of an eyespot may be an example of the uncoupling rule, in which different sub-elements may behave independently [10,24,40]. 

### 4.3. Three Essential Damage Effects

**Figure 7:**
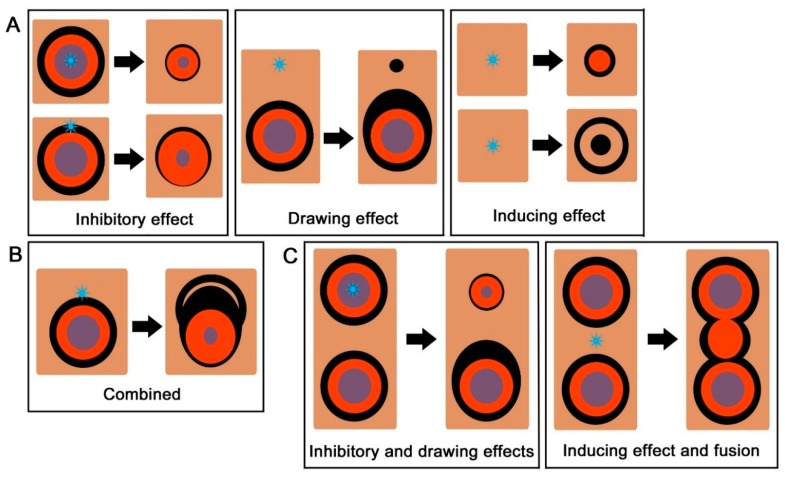
Summary of the damage-induced color pattern modifications. The damage sites are indicated by blue stars. (**A**) Three essential effects: inhibitory, drawing (attracting), and inducing effects. (**B**) Modifications with the three essential effects combined. (**C**) Responses of the anterior and posterior eyespots to a single damage.

The effects of damage on color pattern modifications that were observed in the *J. orithya* hindwing in the present study may be classified as one of the following three types: (1) an inhibitory effect on the entire eyespot or the eyespot core disk (when damage was made at the core disk or the outer rings), (2) a drawing (attracting) effect on the outer black ring (when damage was made outside or in proximity to the eyespot), and (3) an inducing effect for ectopic elements on the background area (when damage was made at the background area) (Figure 7A). Additionally, fusion was observed between the induced element and the innate eyespots (especially when damage was made at the background area between the two eyespots); however, fusion is not likely a phenomenon that was directly elicited by damage but a secondary phenomenon in which the induced and innate elements interacted with each other. Additionally, fusion is probably mechanistically similar to the drawing effect.

Among the three damage effects, the most fundamental response may be the inducing effect because it is observed in the background area without any interactions with elemental signals. Whether the inhibitory or drawing effect occurs may be dependent on the distance between the induced and innate signals. When the two are relatively close, they inhibit each other, and when the two are relatively far away, they attract each other, resulting in the drawing effect or elemental fusion. Homophilic interactions (drawing effect) and heterophobic interactions (inhibitory effect) may be two principles of signal interactions [10,24], but they may also depend on distance.

Interestingly, the three essential effects can be combined in a single case (Figure 7B), suggesting that the three effects are elicited from a coherent morphogenic signal but act independently. Other combinations are also observed when two eyespots are considered simultaneously (Figure 7C); when the anterior eyespot was damaged and thus reduced in size, the posterior eyespot expressed the drawing effect. Additionally, when an ectopic eyespot was induced in the middle of the two eyespots, the induced eyespot fused smoothly with the innate eyespots, indicating that the inducing effect and fusion occurred simultaneously. 

### 4.4. Compromised PFE after Damage

In this study, cPFE was obtained (Figure 2B). This is an important example of direct evidence for the unity of the border symmetry system, meaning that PFEs and eyespots belong to the same system. That is, PFE is the remote “outermost ring” of an eyespot [32,33]. It should be noted that PFE did not respond to eyespot damage in previous studies despite the facts that PFE was dislocated and even absorbed to the nearest eyespot in temperature-shock or chemical treatments [6,15,16,17,18,19,20] and that PFE has been considered a part of the border symmetry system together with eyespot based on local tungstate treatment [32] and color pattern analysis [31,33]. This PFE paradox has been explained by the heterochronic nature of signal release; the PFE signal is released before the eyespot signal is released, and the signals have a wave-like self-progressing nature once released [24,31,33,36]. The cPFE that responded to the physical damage in the present study is thus surprising. Although this is just a single case, it probably means that the release timing of the PFE signal is somewhat variable among individuals, and this case supports the current explanation for the PFE paradox, confirming the status of the PFE as a part of the border symmetry system.

### 4.5. Distortion-Induced Changes

A small stainless ball was placed proximally to the anterior eyespot using the forewing-lift method, and a drawing effect toward the ball placement site was observed (Figure 5A–C), which was fundamentally similar to the damage results. The fact that the ball placement and needle damage resulted in similar phenotypes is important, because it suggests that tissue distortion (instead of puncture damage) is sufficient to influence eyespot size and shape. The ball placement results here are consistent with those of *J. almana* [39].

When the ball was placed more proximally, eyespots were enlarged with or without deformation in many cases (Figure 5D–I). Surprisingly, wing-wide modifications of both anterior and posterior eyespots were also observed by transient forewing-lift operations without ball placement (Figure 6A–F). Thus, it can be concluded that the ball placed at the proximal background area did not contribute significantly to eyespot modification. However, this conclusion does not necessarily mean that distortion does not work at all. Rather, the *J. orithya* wing tissue is so sensitive to distortion that the forewing-lift operation alone can elicit eyespot changes, and the ball could not overcome this “background” level.

In *J. almana*, a transient forewing-lift operation did not induce any modification [39]. The reason for this species difference is not clear, but the hindwing of *J. orithya* is likely more sensitive to subtle physical changes. 

### 4.6. Possible Mechanism of Physical Damage and Candidates for Long-Range Morphogenic Signals

Because physical damage can produce an eyespot-like ectopic element in the background area and because the induced eyespot can fuse smoothly with the innate eyespots (Figure 4), damage-induced signals may be similar to, or even identical to, the innate morphogenic signals in their characteristics. Damage probably not only kills cells, but also introduces distortions in the wing tissue. Hence, ball placement likely causes similar outcomes.

In butterfly wing color pattern systems, some candidate morphogens or their related molecules have been identified [41,42,43,44,45,46,47,48,49,50,51,52]; for example, *Wnt* has been studied intensively. The spatial mRNA distribution patterns of *Wnt* family genes in the pupal wing tissues match nicely to many color pattern elements expressed in adult wings [48,51]. This means that Wnt proteins do not travel a long distance before specifying colors, which makes them unlikely candidates for long-range signals that were posited in this study, although they certainly play an important role in color pattern formation. 

It has been demonstrated that physical damage induces calcium oscillation and waves around the damage site in the pupal wing tissue [53] and that physical damage upregulates at least a few genes that are likely responsible for eyespot formation [41]. Moreover, genes for wound healing and calcium signaling have been detected in prospective eyespot tissues in transcriptome analysis [42].

Similarly, spontaneous calcium waves have been detected that propagate from organizers in the pupal wing tissue [53]. Calcium signals travel long distances from the prospective eyespot centers and from damage sites in butterfly wings [53], and calcium signaling genes are expressed in the prospective eyespot [42]. In the distortion hypothesis, the morphogenic signals are physical distortions that induce calcium waves, followed by gene expression changes [24,39], although a causal contribution of calcium signals to color pattern determination has not been demonstrated yet. 

Contributions of mechanical force and the extracellular matrix to morphogenic signal propagation in butterfly wings have been proposed based on the contact-mediated eyespot color pattern changes in *J. almana* [39]. Synergistic interactions between two eyespots or between two innate or damage-induced signals have been revealed in *J. almana* [11]. The present results further establish the long-range interactive nature of eyespot morphogenic signals in butterfly wings. 

However, other possibilities have not been excluded experimentally. For example, damage may activate hemocytes that eliminate cellular debris. A tissue repair process including cell migration and division may also be involved. These cellular responses may influence ectopic color patterns at a damage site.

## 5. Conclusions

The present study examined color pattern responses to physical puncture damage and distortion on the hindwing of *J. orithya*. Inducing, drawing, and inhibitory effects were observed, suggesting that morphogenic signals are long-ranging and interactive. The present results are consistent with the distortion hypothesis, but further studies are necessary to reveal the nature of the morphogenic signals for eyespot color pattern determination in butterflies.
